# Apyrase decreases phage induction and Shiga toxin release from *E. coli* O157:H7 and has a protective effect during infection

**DOI:** 10.1080/19490976.2022.2122667

**Published:** 2022-09-22

**Authors:** Ida Arvidsson, Ashmita Tontanahal, Karl Johansson, Ann-Charlotte Kristoffersson, Sára Kellnerová, Michael Berger, Ulrich Dobrindt, Diana Karpman

**Affiliations:** a Department of Pediatrics, Clinical Sciences Lund, Lund University, Lund, Sweden; b Institute of Hygiene and Medical Microbiology, Medical University of Innsbruck, Innsbruck, Austria; c Institute of Hygiene, University of Münster, Münster, Germany

**Keywords:** Enterohemorrhagic *Escherichia coli*, RecA, Shiga toxin, apyrase, intestine, mouse, ATP

## Abstract

Shiga toxin (Stx)-producing enterohemorrhagic *Escherichia coli* (EHEC) cause gastrointestinal infection and, in severe cases, hemolytic uremic syndrome which may lead to death. There is, to-date, no therapy for this infection. Stx induces ATP release from host cells and ATP signaling mediates its cytotoxic effects. Apyrase cleaves and neutralizes ATP and its effect on Stx and EHEC infection was therefore investigated. Apyrase decreased bacterial RecA and dose-dependently decreased toxin release from *E. coli* O157:H7 *in vitro*, demonstrated by reduced phage DNA and protein levels. The effect was investigated in a mouse model of *E. coli* O157:H7 infection. BALB/c mice infected with Stx2-producing *E. coli* O157:H7 were treated with apyrase intraperitoneally, on days 0 and 2 post-infection, and monitored for 11 days. Apyrase-treated mice developed disease two days later than untreated mice. Untreated infected mice lost significantly more weight than those treated with apyrase. Apyrase-treated mice exhibited less colonic goblet cell depletion and apoptotic cells, as well as lower fecal ATP and Stx2, compared to untreated mice. Apyrase also decreased platelet aggregation induced by co-incubation of human platelet-rich-plasma with Stx2 and *E. coli* O157 lipopolysaccharide in the presence of collagen. Thus, apyrase had multiple protective effects, reducing RecA levels, *stx2* and toxin release from EHEC, reducing fecal Stx2 and protecting mouse intestinal cells, as well as decreasing platelet activation, and could thereby delay the development of disease.

## Introduction

Enterohemorrhagic *Escherichia coli* (EHEC) cause diarrhea, hemorrhagic colitis, and in certain cases the severe complication hemolytic uremic syndrome (HUS)^1^ characterized by nonimmune hemolytic anemia, thrombocytopenia, and acute kidney injury with up to 5% mortality. There are no specific or effective treatments for this infection and antibiotic treatment, during the prodromal pre-HUS phase, may increase the risk of developing HUS.^2^ *E. coli* O157:H7 is the most common clinical isolate of EHEC.^1^ EHEC infects via oral intake of contaminated food or water and is a noninvasive bacterium.^3^ After ingestion EHEC colonize the intestine.^4^ In the intestine, EHEC release virulence factors such as Shiga toxin (Stx).^5^ During human EHEC infection Stx can be found within intestinal cells^6^ and massive intestinal inflammation and apoptosis have been reported.^7^ Similarly, mice inoculated intragastrically with EHEC exhibit goblet cell depletion,^8^ intestinal inflammation, and apoptosis particularly associated with the presence of Stx.^7^

Stx is encoded by a lambdoid prophage integrated in the bacterial chromosome (reviewed by Rodríguez-Rubio, *et al*.).^9^ The expression of the prophage late genes, including the *stx* determinant, is under the control of the repressor CI.^10,11^ When the SOS response is activated, by various physical or chemical stimuli, as well as by quorum sensing,^12^ the bacterial protein RecA is polymerized and promotes auto-cleavage of the phage repressor CI, and the phage enters the lytic growth cycle inducing *stx* gene transcription.^13^ RecA thereby induces Stx bacteriophage activation ultimately leading to bacterial expression and release of Stx.^14^

Our group has previously shown that Stx1 and Stx2 induced the release of ATP from epithelial cells *in vitro* while Stx2 had a similar effect in a mouse model. In the presence of Stx ATP release increased calcium influx, decreased host cell viability and increased extracellular vesicle release *in vitro*.^15^ Circulating extracellular vesicles transport Stx to the kidney where cellular injury is exerted.^16^ These Stx-mediated effects were blocked by the purinergic receptor antagonists NF449 and suramin,^15^ indicating that Stx caused cellular damage via secondary ATP signaling. During intestinal infection, ATP can be released from both host intestinal cells^17^ and intestinal bacteria.^17,18^ The presence of extracellular ATP in the intestine can contribute to intestinal inflammation and apoptosis.^19^

The ectonucleoside apyrase/CD39 hydrolyses ATP to ADP and ADP to AMP.^20^ In this study, the aim was to investigate whether administration of apyrase, which would eliminate extracellular ATP, could have a beneficial effect during *E. coli* O157:H7 infection using bacterial cultures and an established mouse model.^21^ The effect of apyrase on induction of the bacteriophage, as reflected by RecA levels, *stx2* gene levels and toxin release from *E. coli* O157:H7, were demonstrated *in vitro*. Infected mice treated with apyrase, or left untreated, were assessed for clinical signs of disease, weight loss, goblet cell depletion, intestinal apoptosis as well as the release of fecal ATP and Stx2. Additionally, the effect of apyrase on collagen-induced platelet aggregation in human plasma, in the presence of Stx2 and *E. coli* O157 lipopolysaccharide (O157 LPS), was investigated.

## Results

### Decrease in RecA in E. coli O157:H7 incubated with apyrase

**Figure 1.**
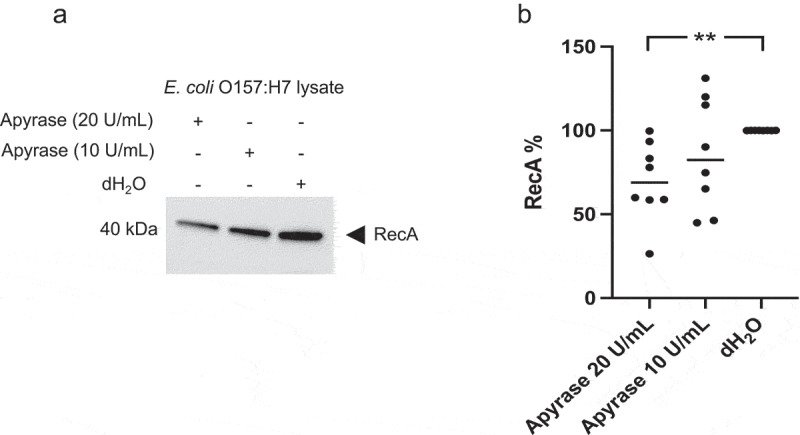
RecA levels in *E. coli* O157:H7 incubated with apyrase 10 and 20 U/mL. **a)** Immunoblot showing RecA in *E. coli* O157:H7 lysate incubated with apyrase for 6 h. **b)** Intensity of RecA bands in relation to dH_2_O control defined as 100%. Data are presented as the median and individual values from two independent experiments, **P < 0.01, Kruskal-Wallis multiple-comparison test followed by Dunn’s procedure.

The level of RecA was investigated as a measure of the SOS response that can lead to bacteriophage activation. *E. coli* O157:H7 was incubated with or without apyrase for 6 h and bacterial lysates were assayed. Lower levels of RecA were detected in samples incubated with apyrase, Figure 1a. A significant difference was detected when measuring the intensity of the RecA band in the *E. coli* O157:H7 sample incubated with apyrase 20 U/mL (median 68.9%) compared to dH_2_O (defined as 100%, Figure 1b and Supplementary Figure 1). Lower levels of RecA were also seen in samples incubated with apyrase 10 U/mL (median 82.5%) but did not achieve statistical significance.

## Decreased induction of the *stx2* prophage in *E. coli* O157:H7 incubated with apyrase

**Figure 2.**
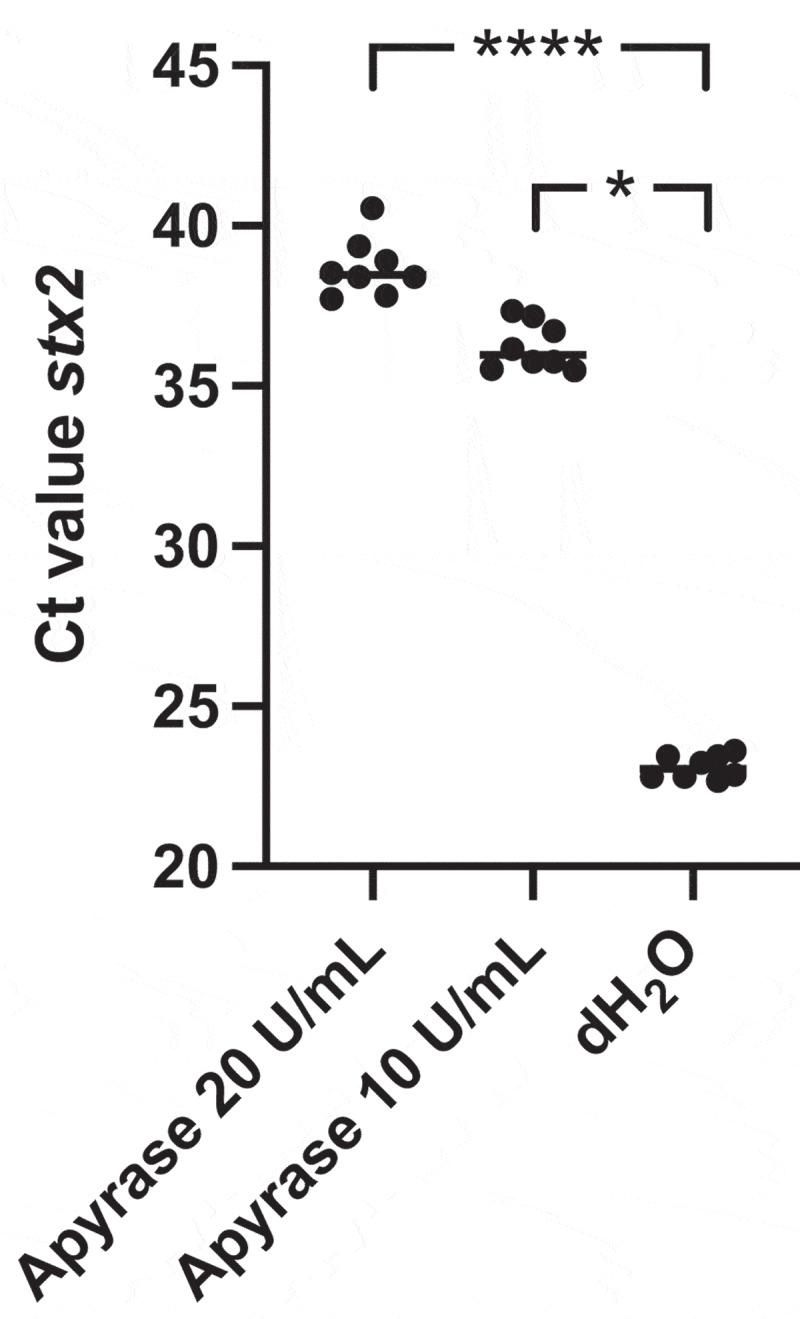
Shiga toxin 2 phage induction in *E. coli* O157:H7 incubated with apyrase *in vitro*. The levels of Stx2 phage DNA *stx2* were measured by qPCR in *E. coli* O157:H7 supernatants from bacteria cultured with or without apyrase (10 or 20 U/mL) for 6 h. The *stx2* gene was quantified as a marker for the Stx2 phage. Data are presented as the median and individual values from two independent experiments, *P < 0.05, ****P < 0.0001, Kruskal-Wallis multiple-comparison test followed by Dunn’s procedure.

To study the impact of apyrase treatment on *stx2* prophage induction, Stx2 phage DNA levels were quantified in *E. coli* O157:H7 supernatants after incubation with or without apyrase for 6 h by qPCR. The *stx2* gene was amplified as a marker for the Stx2 phage. Apyrase decreased the amount of phage DNA in a dose-dependent manner, Figure 2. Phage DNA levels were significantly lower in the presence of apyrase 20 U/mL (median Ct 39) and apyrase 10 U/mL (median Ct 36) compared to the untreated samples (median Ct 23).

## Apyrase decreased Shiga toxin 2 release from *E. coli* O157:H7 *in vitro*

**Figure 3.**
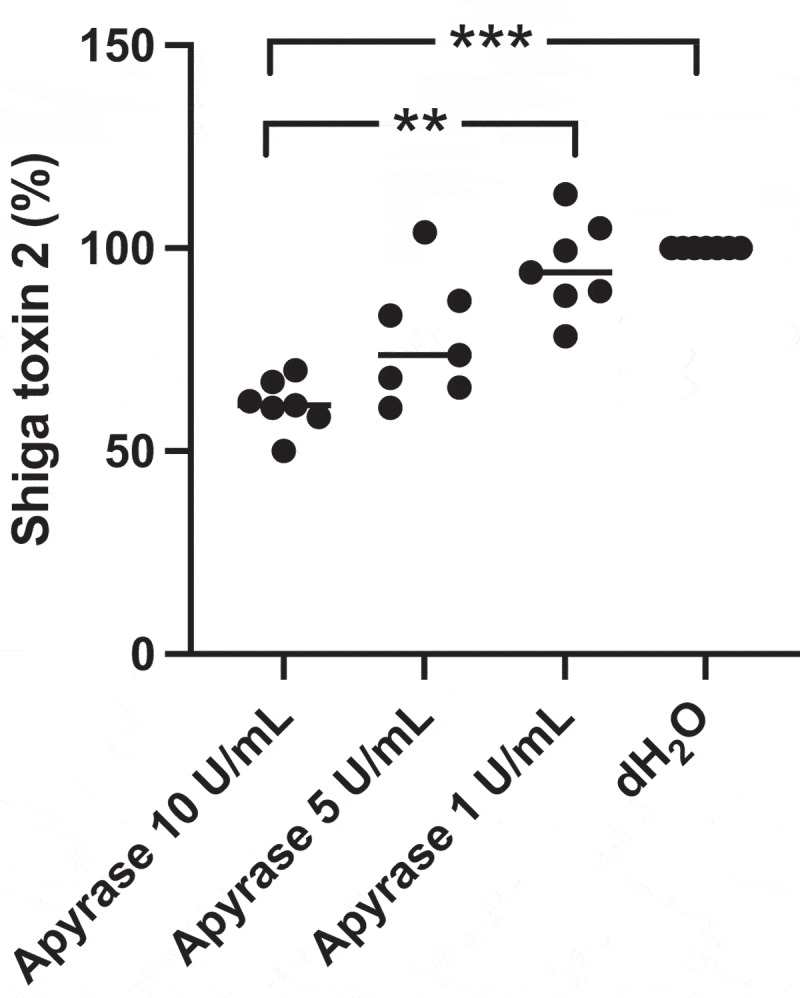
Shiga toxin 2 release from *E. coli* O157:H7 incubated with apyrase *in vitro*. *E. coli* O157:H7 was incubated with 1, 5 or 10 U/mL of apyrase and Stx2 levels measured in culture supernatants. After 6 h incubation there was a dose-dependent and significant decrease in Stx2 release from *E. coli* O157:H7 compared to untreated bacteria. Stx2 concentration from three separate experiments is presented as percentage of the negative control (dH_2_O). The bar represents the median. **P < 0.01, ***P < 0.001, Kruskal-Wallis multiple-comparison test followed by Dunn’s procedure.

*E. coli* O157:H7 was incubated with apyrase for 6 h *in vitro* and the culture supernatants were measured for Stx2 levels. Apyrase decreased Stx2 levels in a dose-dependent manner, as Stx2 concentration was significantly lower after treatment with apyrase at 10 U/mL (median 61% of untreated) compared to apyrase 1 U/mL (median 94% of untreated) and to the untreated (dH_2_O) sample, defined as 100%, Figure 3. The toxin concentrations are presented in Supplementary Figure 2.

## Effect of apyrase treatment on *E. coli* O157:H7 infection

**Figure 4.**
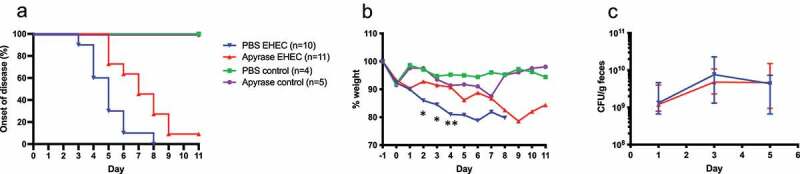
The effect of apyrase treatment on onset of disease, weight and bacterial colonization in mice infected with *E. coli* O157:H7 and uninfected. **a)** Development of clinical signs of disease in *E. coli* O157:H7 (EHEC)-infected mice treated with phosphate-buffered saline (PBS vehicle, n = 10, blue line), apyrase (n = 11, red line) and uninfected mice treated with PBS (vehicle, n = 4, green line) or apyrase (n = 5, purple line). Infected mice in the untreated group started to develop symptoms from day 3 whereas mice in the apyrase-treated group started to develop symptoms 2 days later. **b)** Weight changes in mice starting 1 day before inoculation with EHEC until day 11 post-inoculation. A significant difference between infected groups treated with apyrase or PBS was observed on day 2 and 3 (both *P < 0.05) and day 4 **P < 0.01, two-tailed Mann-Whitney U test. **c)** Colony forming units in feces of EHEC-infected mice, treated with apyrase or untreated, on days 1, 3 and 5 showing no difference in colonization. Data are presented as median and range.

The effect of apyrase treatment was assessed in a previously described *E. coli* O157:H7 infection mouse model.^21^ Mice infected with *E. coli* O157:H7 and left untreated (given vehicle instead of apyrase treatment, n = 10) developed symptoms from day 3 onwards and were sacrificed either upon the development of symptoms or weight loss ≥ 20%. In contrast, the group infected with *E. coli* O157:H7 and treated with apyrase (n = 11) developed symptoms two days later, on days 5–9, with a significant difference in the time of disease onset between the two infected groups P < 0.01, log-rank test, Figure 4a.

Mouse weight was monitored daily, and weight loss was noted in both groups inoculated with *E. coli* O157:H7, Figure 4b. The earliest and the most pronounced weight loss was observed in the group infected with *E. coli* O157:H7 and left untreated. A significant difference between the groups infected with *E. coli* O157:H7 and treated with apyrase or left untreated was observed on days 2, 3 and 4.

Bacterial colony forming units (CFU) were analyzed on day 1, 3 and 5 after inoculation with *E. coli* O157:H7. No significant difference in fecal EHEC levels was observed between the mice treated with apyrase or left untreated, Figure 4c.

In a separate set of experiments, mice were sacrificed on day two, to obtain samples at a specific time point, before the development of disease. These included *E. coli* O157:H7-infected mice that were untreated (n = 12) or treated with apyrase (n = 12) and uninfected mice that were not treated (n = 7) or treated with apyrase (n = 8). No difference in weight or colony forming units (in infected mice) was observed between these groups (Supplementary Figure 3). Intestines from these mice were used for visualization of goblet cells and apoptotic cells using the terminal deoxynucleotidyl transferase dUTP nick end labeling (TUNEL) assay described below.

## Apyrase protected goblet cells from mucus depletion

**Figure 5.**
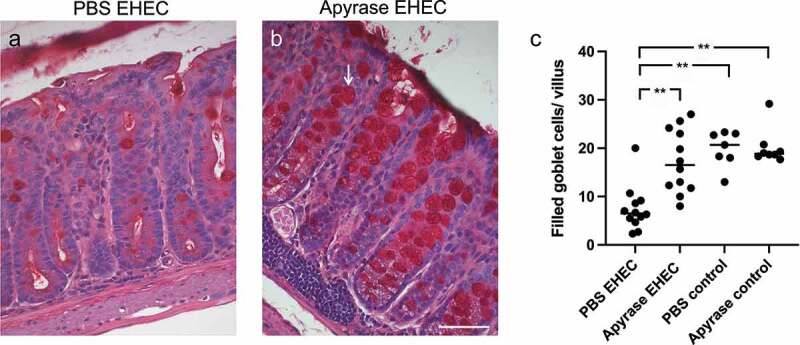
The effect of apyrase on goblet cell depletion in mice infected with *E. coli* O157:H7. Goblet cells in Periodic acid-Schiff (PAS)-stained colons from mice sacrificed 2 days after inoculation with *E. coli* O157:H7 before the development of symptoms. **a)** An *E. coli* O157:H7-infected and vehicle-treated mouse exhibited few filled goblet cells. **b)** Colon from a mouse infected with *E. coli* O157:H7 and treated with apyrase showed an abundance of filled goblet cells (arrow). **c)** The number of filled goblet cells/villus in *E. coli* O157:H7-infected mice treated with vehicle (PBS) (n = 12), or apyrase (n = 12) or uninfected controls treated with vehicle (n = 7) or apyrase (n = 8). Filled goblet cells per villus are presented from individual mice, the bar represents the median. **P < 0.01, Kruskal-Wallis multiple-comparison test followed by Dunn’s procedure. Scale bar 50 μm.

Mice infected with *E. coli* O157:H7 and sacrificed on day 2 showed differences in the number of mucus-filled goblet cells. Colons from *E. coli* O157:H7-infected mice left untreated showed only few filled goblet cells, Figure 5a. Mice infected and treated with apyrase showed more mucin-positive filled goblet cells, Figure 5b. A lower number of filled goblet cells were found in the group infected with *E. coli* O157:H7 and left untreated (median 6.5 filled goblet cells/villus) compared to mice infected with *E. coli* O157:H7 and treated with apyrase (median 16.5 filled goblet cells/villus), P < 0.01, Figure 5c. The uninfected controls showed no mucus depletion (vehicle, median 20.6 filled goblet cells/villus and apyrase, median 18.3 filled goblet cells/villus).

## Apyrase treatment protected intestinal cells from apoptosis

**Figure 6.**
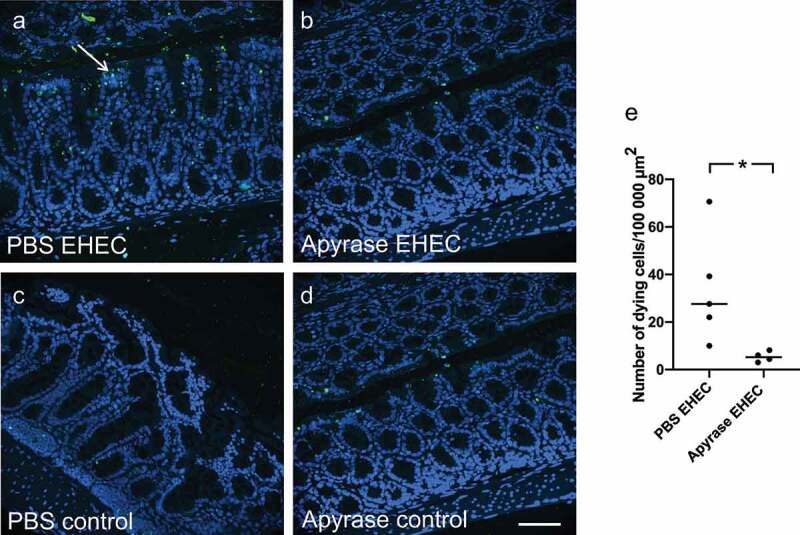
Intestinal apoptosis in mice infected with *E. coli* O157:H7. TUNEL-positive cells were assessed in mouse colons 2 days after inoculation with *E. coli* O157:H7 before the development of symptoms. **a)** Colon from a mouse inoculated with *E. coli* O157:H7 and treated with vehicle exhibited apoptotic cells (white arrow, green labeling) close to the lumen. **b)** Colon from an infected mouse treated with apyrase showed minimal TUNEL-positivity. **c)** Uninfected control mouse treated with vehicle. **d)** Uninfected control mouse treated with apyrase. **e)** Number of TUNEL-positive cells/100,000 μm^2^ in *E. coli* O157:H7-infected mice treated with vehicle (PBS) (n = 5) or apyrase (n = 4). The bar represents the median. Green: TUNEL positive cells; blue: DAPI: 4',6-diamidino-2-phenylindole. * P < 0.05, two-tailed Mann-Whitney U test. Scale bar 100 μm.

The number of apoptotic cells in intestinal sections from mice sacrificed on day 2 was assessed by the TUNEL assay. Apoptotic cells were observed on the luminal side of the intestine, Figure 6. Staining was also observed in lymphatic tissue both in infected and uninfected mice. The number of dying cells was quantified and is presented as the number of TUNEL-positive cells/100 000 μm^2^. The highest number of apoptotic cells was observed in the group infected with *E. coli* O157:H7 and left untreated, Figure 6a. Few TUNEL-positive cells were found in mice infected with *E. coli* O157:H7 and treated with apyrase (Figure 6b) and in uninfected controls that were untreated (Figure 6c) or treated with apyrase, Figure 6d. A higher number of apoptotic cells was observed in the intestines of the mice infected with *E. coli* O157:H7 and left untreated (median 27.6, range 10–70.6 cells/100 000 μm^2^) compared to mice infected and treated with apyrase (median 5.2, range 3–8.2 cells/100 000 μm^2^) P < 0.05, Figure 6e. The uninfected controls showed minimal TUNEL-positive cells (vehicle, median 1.1, range 0–5 cells/100 000 μm^2^ and apyrase-treated, median 3.6, range 1–14.8 cells/100 000 μm^2^).

## ATP in feces from *E. coli* O157:H7-infected mice

**Figure 7.**
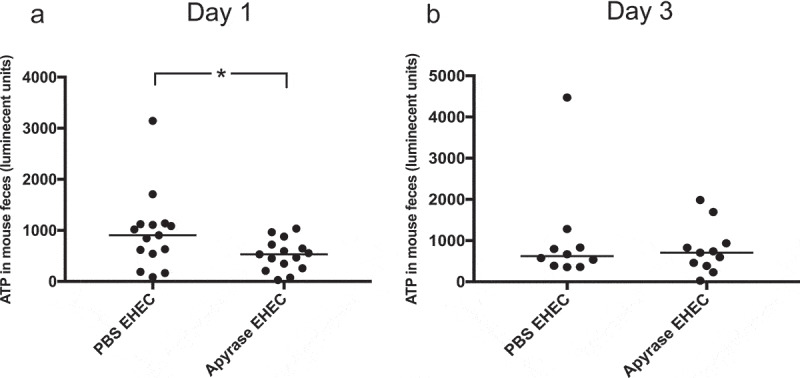
ATP in feces of *E. coli* O157:H7-infected mice. Levels of ATP in mouse feces were assayed. **a)** One day after *E. coli* O157:H7 infection, in PBS-treated (n= 15) or apyrase-treated mice (n= 15), showing significantly lower ATP in the apyrase-treated mice. **b)** ATP in murine feces three days after EHEC infection in mice treated with PBS (n=10) or apyrase (n=11), showing no difference between the groups. Luminescence units in candela per square meter (cd/m_2_). Data are presented from individual mice, the bar represents the median. *P<0.05, two-tailed Mann-Whitney U test.

Fecal samples taken one day after *E. coli* O157:H7 infection showed higher levels of ATP in mice left untreated (median 904, luminescent units) compared to mice treated with apyrase (median 533), P < 0.05, Figure 7a. No significant difference was found comparing the different infected groups on day 3, Figure 7b.

## Shiga toxin 2 in feces from *E. coli* O157:H7-infected mice

**Figure 8.**
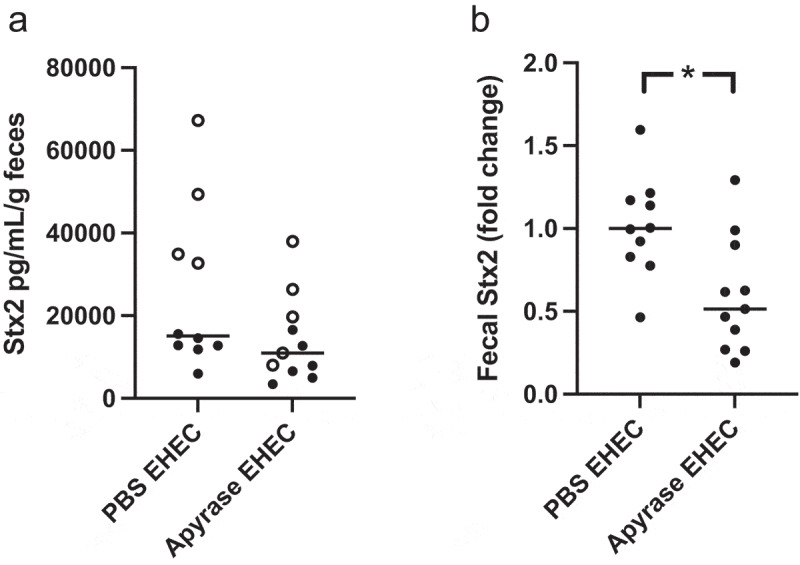
Shiga toxin 2 in fecal samples from *E. coli* O157:H7-infected mice. Levels of Shiga toxin 2 (Stx2) in murine feces were analyzed. **a)** Stx2 in feces three days after *E. coli* O157:H7 inoculation in PBS vehicle-treated (n=10) or apyrase-treated (n=11) mice. Two separate experiments are presented in which unfilled and filled circles represent one experiment each. **b)** Fold change of Stx2 in feces three days after *E. coli* O157:H7 infection. The median of PBS vehicle-treated mice was defined as 1. *P<0.05, two-tailed Mann Whitney U test. Data are presented from individual mice, the bar represents the median.

Fecal samples taken on day 1 and 3 after bacterial inoculation were analyzed for Stx2 concentrations. On Day 1 there was no difference in fecal Stx2 (untreated (n = 22 mice) median 12014 pg/mL/g feces, range 4175–39266, apyrase-treated (n = 23 mice) median 12758 pg/mL/g feces, range 2765–27153). On Day 3 after inoculation fecal Stx2 was lower in the apyrase-treated group, Figure 8a shows absolute levels and Figure 8b shows fold change.

## Apyrase decreased platelet aggregation induced in the presence of Shiga toxin 2 and O157 LPS

**Figure 9.**
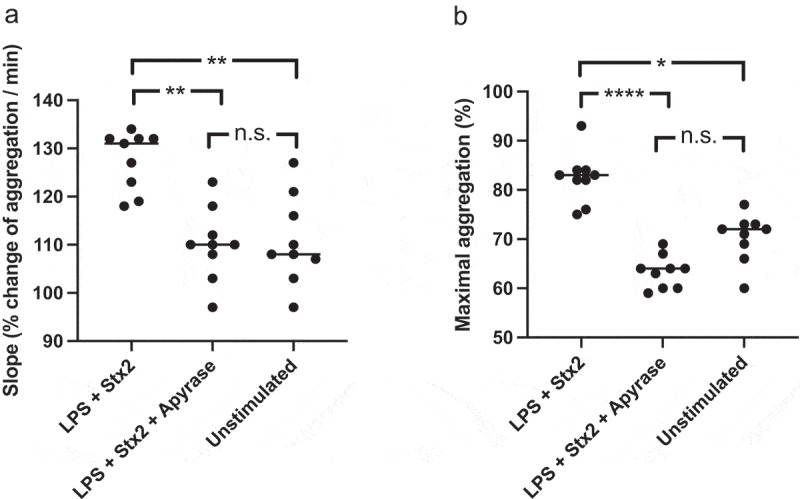
Aggregation in platelet-rich-plasma treated with apyrase and stimulated with Shiga toxin 2 and O157 LPS. Platelet aggregation induced by collagen in platelet-rich-plasma preincubated with Shiga toxin (Stx2) and *E. coli* O157 lipopolysaccharide (LPS) or left unstimulated (PBS), with or without apyrase. **a)** The speed of platelet aggregation (slope, see Supplementary Figure 4) in platelet-rich-plasma preincubated with Stx2 and O157 LPS was increased compared to unstimulated collagen-activated platelets. In the presence of apyrase this increase was abrogated. **b)** Similar differences were observed in maximal platelet aggregation with higher aggregation in samples preincubated with Stx2 and O157 LPS compared to untreated samples. Apyrase abolished this effect. Data are presented from individual experiments, the bar represents the median. n.s: not significant, *P < 0.05, **P < 0.01, ****P < 0.0001. Kruskal-Wallis multiple-comparison test followed by Dunn’s procedure.

Human platelets preincubated with Stx2 and O157 LPS aggregated significantly faster and more when activated with collagen compared to unstimulated collagen-activated platelets, Figure 9 and Supplementary Figure 4. Platelets stimulated with Stx2 and O157 LPS, followed by collagen activation, exhibited significantly lower aggregation when treated with apyrase 1 U/mL, as demonstrated by the aggregation slope (median 110% change aggregation/min) (Figure 9a) compared to untreated (median 131% change aggregation/min). The same tendency was seen when analyzing maximal aggregation, in the presence of apyrase the median was 64% compared to untreated platelets, median 83% (Figure 9b). Apyrase alone at 1 U/mL did not inhibit collagen-induced platelet aggregation (Supplementary Figure 5).

## Apyrase did not protect cells from Shiga toxin 2-induced cytotoxicity

HeLa cells, HUVEC, or pGEC were incubated separately with Stx2 for 24 h. Cell viability was affected in all cells when incubated with Stx2 at 10 ng/mL (HeLa cells 27% viability, HUVEC 81% and pGEC 26%) or 50 ng/mL (HeLa cells 16%, HUVEC 67% and pGEC 11%). Co-incubation with apyrase did not protect the cells and incubation with apyrase alone did not affect cell viability (Supplementary Figure 6).

## Discussion

Stx is the main virulence factor of *E. coli* O157:H7. In the most severe cases *E. coli* O157:H7 infection leads to hemolytic uremic syndrome and an increased risk of death. Stx stimulates ATP release and secondary ATP signaling via purinergic receptors contributes to host cell activation and Stx-mediated cytotoxicity.^15^ In this study ATP/ADP phosphohydrolysis by apyrase was shown to decrease RecA protein levels as well as Stx2 prophage induction and thereby reduced Stx2 release from *E. coli* O157:H7 *in vitro*. This effect would potentially decrease the toxin load reaching target organs and thereby delay disease onset. This was assessed in an *E. coli* O157:H7 mouse model in which apyrase was shown to decrease fecal Stx2, prevent goblet cell depletion, protect intestinal cells from apoptosis and delay systemic symptoms. Not only did apyrase protect host cells in the intestine by decreasing extracellular ATP, it also exhibited an inhibitory effect on collagen-induced platelet aggregation using platelets that were activated with Stx2 and O157 LPS. The results suggest that apyrase exerts an effect on the bacterium *E. coli* O157:H7 by diminishing toxin release and a protective effect on the host by reducing intestinal injury and platelet aggregation.

Extracellular ATP may originate from host cells and from intestinal bacteria.^17^ Apyrase-induced ATP hydrolysis was shown to affect RecA levels and Stx release from the bacteria. By reducing RecA levels the SOS response is decreased. RecA activation promotes autocleavage of the phage repressor CI and less RecA will thereby lead to a decrease in Stx release.^14^ This can explain how apyrase lowered Stx2 toxin levels *in vitro*. In line with this, apyrase was shown to decrease Stx2 prophage induction. *E. coli* O157:H7 is a noninvasive strain and after it colonizes the intestine the toxin gains access to the circulation. Toxin circulates, to a certain extent, within host cell-derived extracellular vesicles,^16^ and is taken up by kidney cells, leading to renal failure. If less SOS response is induced in the colonizing EHEC population, the fraction of Stx phage-mediated bacterial lysis and Stx release will be lower, as reflected by lower fecal Stx2 levels.

ATP is released from cells during intestinal infection and inflammation.^22^ ATP removal by apyrase decreases apoptosis, as shown in systemic endotoxemia^23^ and in the intestines from infected mice studied herein. Induction of murine intestinal inflammation and colitis, by feeding with dextran sulfate^24^ or systemic administration of LPS^23^ was strongly associated with proinflammatory signaling as demonstrated by inflammasome activation, cytokine production, and intestinal barrier disruption, effects that were mediated by extracellular ATP and blocked by apyrase or P2X7 receptor antagonism.^23–25^ Dephosphorylation of extracellular ATP to adenosine is associated with anti-inflammatory effects in the intestine.^22^ Our previous study showed that purinergic receptor blockade, or P2X1 silencing, diminished cellular caspase 3/7 activation by Stx1 *in vitro*.^15^ Taken together, removal of extracellular ATP and its signaling, as shown herein by decreased fecal ATP and Stx2 levels in apyrase-treated mice, may have a protective effect in the mouse model of EHEC infection by upholding the intestinal barrier (goblet cell secretion of mucus) and reducing intestinal cell apoptosis. This contributed to the delay in development of disease in apyrase-treated mice. Daily treatment with apyrase for a longer time-period than 2 days post-inoculation with *E. coli* O157:H7 might be even more protective.

Once Stx gains access to the systemic circulation it exerts its effects by inducing thrombotic microangiopathy. This lesion is achieved by endothelial cell damage^26^ as well as by platelet activation.^27^ The latter effect of Stx only occurs when platelets are preactivated,^28^ such as in the presence of LPS.^29^ We therefore investigated the effect of apyrase treatment on platelet aggregation induced by collagen when platelets were activated by co-stimulation with Stx2 and O157 LPS. Exogenous ATP signals via P2X1 ion channels on platelets, thereby inducing platelet activation.^30^ We found that Stx2 together with LPS from *E. coli* O157:H7 induced increased platelet aggregation compared to unstimulated collagen-treated platelets. In the presence of apyrase the effect of Stx2/O157 LPS on enhanced platelet aggregation was abrogated. Thus, apyrase exhibits an effect on platelet activation that could inhibit the development of thrombotic lesions.

Removal of extracellular ATP with apyrase has been suggested as a treatment for systemic inflammation^23^ and infection. Apyrase treatment reduced biofilm formation, and in burns reduced neutrophil infiltration and necrosis.^31^ Apyrase eradicated *Acinetobacter baumannii* infection while stimulating wound healing.^32^ Apyrase or apyrase-like agents have been proposed for the treatment of infections such as sepsis, wounds, and burns. A patent application (submitted by APT Therapeutics) mentions that the administration route could be oral, topical, inhalatory, subcutaneous, intramuscular, or parenteral.^33^ In the present study, apyrase was administered intraperitoneally. Intraperitoneal administration of apyrase protected mice from ischemia-reperfusion-induced renal injury.^34^ Intraperitoneal administration is not a feasible approach during human gastrointestinal EHEC infection but oral administration or parenteral injection could be investigated. Using *in vivo* models, apyrase has been studied for multiple indications, including reduction of clot formation,^35^ but, to-date, human trials have not been reported.

In summary, this study demonstrates that removal of extracellular ATP by apyrase decreased Stx release from *E. coli* O157:H7 and delayed the development of disease in EHEC-infected mice. Apyrase had a protective effect on the host intestine by reducing goblet cell mucin depletion and intestinal cell apoptosis. It also reduced the combined effect of Stx2 and O157 LPS on platelet aggregation. Apyrase thereby exerted a protective effect by acting on multiple targets, both on the bacterium and on the host. Administration of apyrase or an apyrase-like agent during EHEC infection should therefore be further evaluated as a treatment option.

## Material and methods

### Bacterial strain and cultures

The Stx2-producing *E. coli* O157:H7 strain 86**–**24 (kindly provided by A. D. O’Brien, Uniformed Services University of the Health Sciences, Bethesda, MD) was previously characterized.^7^ A spontaneously obtained streptomycin-resistant derivate of this strain was used.^21^

## *E. coli* O157:H7 supernatants and lysates

*E. coli* O157:H7 was grown overnight at 37°C in LB-broth supplemented with streptomycin (50 μg/mL). The overnight culture was centrifuged at 1500 g for 10 min at room temperature (RT), the supernatant was removed, and the pellet was resuspended and diluted 1:1000 in LB-broth containing streptomycin. The bacterial suspension was combined with potato apyrase grade VII (Sigma-Aldrich, Steinheim, Germany) 1, 5, 10, or 20 U/mL diluted in dH_2_O and incubated for 6 h at 37°C. The optical density at 600 nm was measured (no difference in growth was observed, Supplementary Figure 7) and the samples were centrifuged 1500 g for 10 min followed by 13 000 g for 3 min at RT. The supernatants were filtered (0.2 µm, Pall, Ann, Arbor, MI) and stored at −80°C and used for detection of Stx2. Protein lysates from the pellets were prepared using RNAprotect® bacteria reagent (Qiagen, Hilden, Germany) followed by lysis with Tris-EDTA buffer (Sigma) containing lysozyme (1 mg/mL, Sigma) for 5 min at RT. The whole cell lysates were analyzed for RecA by immunoblotting.

## Detection of RecA in *E. coli* O157:H7 lysates

*E. coli* O157:H7 lysates were reduced using 2-mercaptoethanol and loaded on a 4–20% mini protean TGX gel (Bio-Rad, Hercules, CA). The proteins were blotted on a PVDF membrane (Bio-Rad) and blocked using casein (Vector laboratories, Burlingame, CA). The membrane was incubated with rabbit anti-RecA, 1:3000 (Abcam) for 1 h at RT. Binding was detected with goat anti-rabbit HRP 1:1000 (Dako) followed by Pierce ECL plus Western Blot substrate (Thermo Fisher Scientific) and visualized with ChemiDoc™ Touch, Bio-Rad. The band intensity was measured using Image Lab 6.0.1, Bio-Rad.

## Quantification of phage-induced plaques and the *stx2* gene as a marker of phage induction

To quantify viable phages a plaque assay was performed as previously described^36^ using culture supernatant from *E. coli* O157:H7 (strain 86–24). The plaques obtained were too small to quantify. The levels of the Shiga toxin gene, *stx2*, representing the Stx2 phage, were therefore quantified in culture supernatants, described above, by qPCR, as previously described.^37^ DNA was extracted from 100 μL supernatant using InstaGene matrix (Bio-Rad) according to the manufacturer’s instructions. The *stx2* gene was amplified in a 20 μL reaction buffer containing 1 μL of the extracted DNA, TaqMan Fast Advanced Master Mix (Applied Biosystems, Waltham, MA) primers and probe (Eurofins, Ebersberg, Germany) as described,^37^ using QuantStudio 3 (Applied Biosystems).

## Detection of Shiga toxin 2

Detection of Stx2 in the *E. coli* O157:H7 culture supernatants and in fecal samples from mice, described below, was carried out by ELISA, as previously described.^38^ Briefly, camelid anti-Stx antibody (List Biological Laboratories, Campbell, CA) was coated on a white MaxiSorp nunc plate (Thermo Fisher Scientific, Rockford, IL) and incubated overnight at 4°C. The plate was washed, blocked, and incubated with samples overnight at RT. The plate was washed, incubated with mouse anti-Stx2 (Santa Cruz Biotechnology, Dallas, TX) for 1 h at RT, washed again and incubated with goat anti-mouse IgG-HRP (Dako, Glostrup, Denmark) for 1 h at RT. The unbound antibody was washed away, and the plate was developed using Super signal ELISA pico chemiluminescent substrate (Thermo Fisher Scientific) and detected at one-sec integration time in the Glomax Discover system. Stx2 (Phoenix Lab, Tufts Medical Center, Boston, MA) was used as the standard.

## Mice

BALB/c mice were bred at the Center for Comparative Medicine, Medical Faculty, Lund University. Both female and male mice were used at 8**–**12 weeks of age. Mice were age-matched in each experiment. Two to five mice were kept in individual ventilated cages (Innovive) containing cage enrichment.

## *E. coli* O157:H7-infection mouse model

Mice were infected with *E. coli* O157:H7 according to a previously described infection protocol at a concentration of 10^9^ colony forming units (CFU)/mL diluted in a solution of 20% (w/v) sucrose and 10% (w/v) NaHCO_3_ in water.^21^ Mice were treated with streptomycin (5 g/L) in drinking water 24 h before inoculation with *E. coli* O157:H7 and throughout the experiment to enhance intestinal colonization. Before infection with *E. coli* O157:H7 the mice were fasted for food but not water for 16 h. Each mouse was inoculated with 10^8^ CFU in a volume of 100 μl. Controls were inoculated with the same volume of the sucrose/NaHCO_3_ solution without bacteria. After inoculation food was provided. Mice were monitored two to four times a day. Mice were weighed and observed daily for signs of disease (ruffled fur, lethargy, hunched posture, decreased activity, paralysis, tremor, ataxia, and weight loss ≥20%), as previously described.^21^ Mice were sacrificed at the first sign of disease or, if unaffected, on day 11 after inoculation. Certain mice were sacrificed on day 2 of the experiment before symptoms developed. Fecal samples were collected on days 1, 3 and 5 after inoculation to confirm intestinal colonization and the effect of apyrase. Samples were weighed, dissolved in 1 mL PBS and serial dilutions were carried out and plated on Luria-Bertani agar supplemented with 50 μg/mL streptomycin. Colonies were counted and confirmed to be *E. coli* O157:H7 using a Latex agglutination kit (Oxoid, Basingstoke, U.K.). The remaining fecal sample from each mouse was centrifuged at 1500 g for 10 min, the supernatant was transferred to a new Eppendorf tube and centrifuged at 13 000 g for an additional 5 min. The supernatant was sterile filtered (0.2 μm Pall) and stored at −80°C until analyzed for ATP content and Stx2 levels (described below).

All animal experiments were approved by the animal ethics committee of Lund University (approval number: 17452–20) in accordance to the guidelines of the Swedish National Board of Agriculture and the EU directive for the protection of animals used in science.

## Apyrase treatment

Mice were injected intraperitoneally with apyrase 150 μl, 15 U/mouse on day 0 just before EHEC inoculation and 2 days after inoculation, control mice received the same volume of sterile PBS (vehicle) at the same time points (with or without EHEC inoculation). This dose of apyrase was chosen at it previously was shown to protect mice in an LPS model.^23^ Mice sacrificed on day 2 received two doses of apyrase.

## Imaging and quantification of goblet cells

Mucus-filled intestinal goblet cells have previously been shown to be depleted during EHEC infection in mice.^8^ Colon sections were stained with Periodic acid-Schiff (PAS) staining kit (Abcam, Cambridge, UK) according to the manufacturer’s instructions to visualize mucus in goblet cells. Three colonic regions per mouse containing at least three villi were examined. Images were obtained using a Nikon TiEclipse microscope, equipped with a Nikon color camera and analyzed with NIS elements AR software v.5.11.01, and the number of mucus-filled goblet cells per villus was quantified in blinded fashion.

## TUNEL assay of mouse colons

Intestinal apoptosis is increased in both human and murine EHEC infection.^7^ Mouse colons were stained using the Click-it Plus TUNEL assay (Invitrogen, Eugene, OR) according to the manufacturer’s instructions. The slides were mounted using Prolong diamond antifade with DAPI (Invitrogen) to visualize all nuclei. TUNEL-positive cells were visualized using the fluorescence microscope Nikon TiEclipse equipped with a Hamamatsu flash camera. The number of apoptotic cells was assessed in blinded fashion by detection of TUNEL-positive nuclei in five separate areas, each 100 000 μm^2,^ using ImageJ software (Version 1.48 v, NIH, Bethesda).

## Detection of ATP and Shiga toxin in mouse feces

Mouse feces collected on day 1 and 3 were diluted in PBS at 1 mg/100 μL. Detection of ATP was carried out using a bioluminescence assay. ATP content was analyzed using firefly luciferase (65 nM, Sigma-Aldrich) and D-luciferin (1.3 mM, Invitrogen) with detection at one-sec integration time in a Glomax Discover system (Promega, Madison, WI).

Detection of Stx2 in the fecal samples was carried out using the Stx2 ELISA described above. Stx2 levels were adjusted to the weight of feces in grams.

## Platelet aggregation

Blood was collected in citrated tubes (BD, Franklin Lakes, NJ) from healthy donors. Blood samples were centrifuged at 200 g for 20 min RT. Platelet rich plasma was transferred to a new tube and the remaining blood sample was centrifuged at 2000 g for 10 min to obtain platelet poor plasma. An aggregation assay was performed in an aggregometer with four channels (Chrono-log, model 490 4+). Platelet-rich plasma, 225 μL was incubated for 5 min with 25 μL apyrase at different concentrations (final concentration 1, 5 and 10 U/mL in PBS). After 5 min 25 μL collagen type 1 (final concentration 2 μg/mL, Chrono-log, Havertown, PA) was added and similar aggregation was recorded in the unstimulated sample and that treated with apyrase 1 U/mL, whereas the higher concentrations exhibited reduced platelet aggregation (Supplementary Figure 5). Apyrase at 1 U/mL was therefore used in the following experiments. Platelet-rich plasma, 225 μL incubated with 25 μL PBS alone or combined with Stx2 (final concentration 0.2 μg/mL) and *E. coli* O157 lipopolysaccharide O157 LPS (final concentration 1 μg/mL, a gift from R. Johnson, Public Health Agency, Guelph, ON, Canada) or Stx2, O157 LPS and apyrase for 5 min at 1200 rpm/min 37°C. After 5 min 25 μL collagen type 1 (final concentration 2 μg/mL) was added to the cuvette and aggregation was recorded as light transmission.

Blood samples from healthy subjects were taken with the approval of the Swedish Ethical Review Authority (2021–04438). All samples were taken in accordance with the declaration of Helsinki and with informed written consent of the subjects.

## Cell cultures

HeLa cells (cervical epithelial, a gift from L. Johannes, Institute Curie, Paris) were cultured in DMEM supplemented with 10% fetal calf serum and 1% penicillin-streptavidin (Gibco, Waltham, MA). Primary human umbilical vein endothelial cells (HUVEC, ATCC, Manassas, VA), and primary glomerular endothelial cell (pGEC) (Cell systems, Kirkland, WA) were cultured in EBM-2 bullet kit supplemented with 1% penicillin-streptavidin (Gibco) at 37°C with 5% CO_2_.

## Viability assay

HeLa, HUVEC, and pGEC (10 000 cells/well) were seeded and HUVEC, and pGEC were preincubated with TNF-α (10 ng/mL, Sigma-Aldrich) for 24 h at 37°C. Cells were washed and incubated with or without apyrase (1 U/mL) diluted in medium without fetal calf serum for 30 min at 37°C. Apyrase was removed, replaced with serum-free medium with or without apyrase, at the same concentration, and Stx2 (10 and 50 ng/mL), as well as medium without Stx2, and incubated for 24 h at 37°C. Cell viability was measured using Alamar blue (Thermo Fisher Scientific) according to manufacturer’s instructions and measured using the Glomax Discover system.

## Statistical analysis

Differences between groups was assessed by the two-tailed Mann-Whitney U test or when comparing more than two groups by Kruskal-Wallis multiple-comparison test followed by Dunn’s procedure. Kaplan–Meier curves were analyzed using log-rank test. A P-value ≤0.05 was considered significant. All statistical analyses were calculated using Prism 8 version 8.4.3 (GraphPad, La Jolla, CA).

## Supplementary Material

Supplemental Material

## Data Availability

The authors confirm that the data supporting the findings of this study are available within the article and its supplementary materials.
