# Supplementary material for: Apyrase decreases phage induction and Shiga toxin release from E. coli O157:H7 and has a protective effect during infection
Source: Gut Microbes. 2022 Sep 22;14(1):2122667. doi: 10.1080/19490976.2022.2122667 (PMC9519026; doi:10.1080/19490976.2022.2122667)
Supplement: Supplemental Material [file KGMI_A_2122667_SM5382.zip › Supplementary Figure 3.pdf]

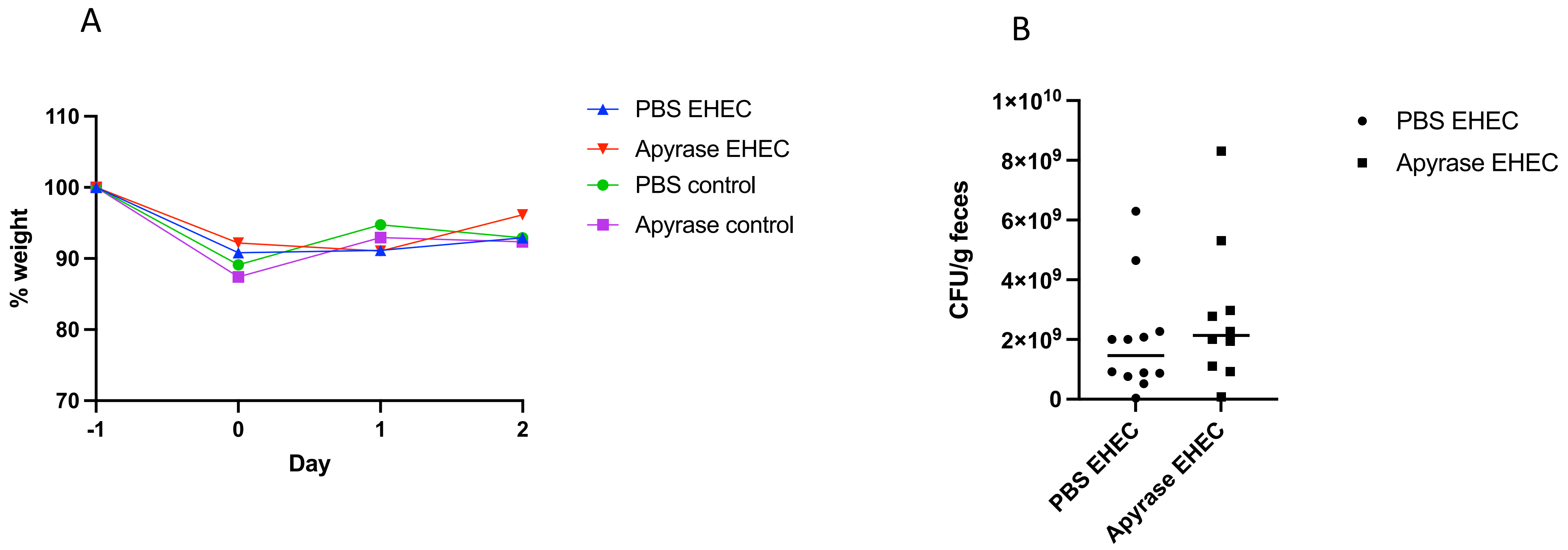

Supplementary Figure 3: The effect of apyrase treatment on weight and bacterial colonization in mice infected with *E. coli* O157:H7 or uninfected until day two.

A) Weight changes in mice starting from day -1, before inoculation with EHEC, until day 2 post-inoculation. **B)** Colony forming units in feces on day 1 in mice sacrificed on day 2.
