# Supplementary material for: Apyrase decreases phage induction and Shiga toxin release from E. coli O157:H7 and has a protective effect during infection
Source: Gut Microbes. 2022 Sep 22;14(1):2122667. doi: 10.1080/19490976.2022.2122667 (PMC9519026; doi:10.1080/19490976.2022.2122667)
Supplement: Supplemental Material [file KGMI_A_2122667_SM5382.zip › Supplementary Figure 6.pdf]

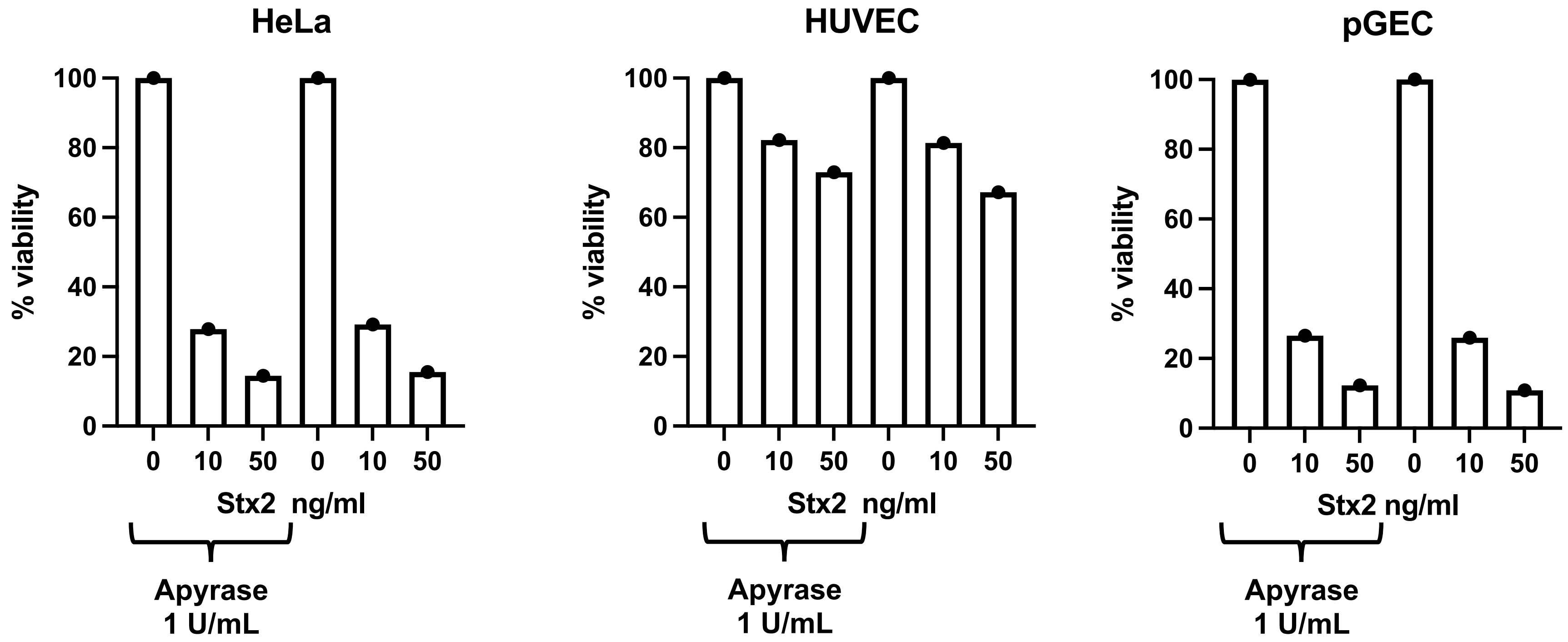

Supplementary Figure 6: Apyrase did not protect cells from Shiga toxin induced toxicity.
HeLa cells, HUVEC (human umblical vein endothelial cells), or pGEC (primary glomerular endothelial cells) incubated with Stx2 alone (0, 10 or 50 ng/mL) or Stx2 with apyrase for 24 h. Cell viability was decreased in the presence of Stx2 in all cells incubated with Stx2 10 and 50 ng/mL and apyrase did not have a protective effect. Viability is presented as percent in which cells without Stx2 were defined as 100 %.
