# Supplementary figures and images for: Apyrase decreases phage induction and Shiga toxin release from E. coli O157:H7 and has a protective effect during infection
Source: Gut Microbes. 2022 Sep 22;14(1):2122667. doi: 10.1080/19490976.2022.2122667 (PMC9519026; doi:10.1080/19490976.2022.2122667)

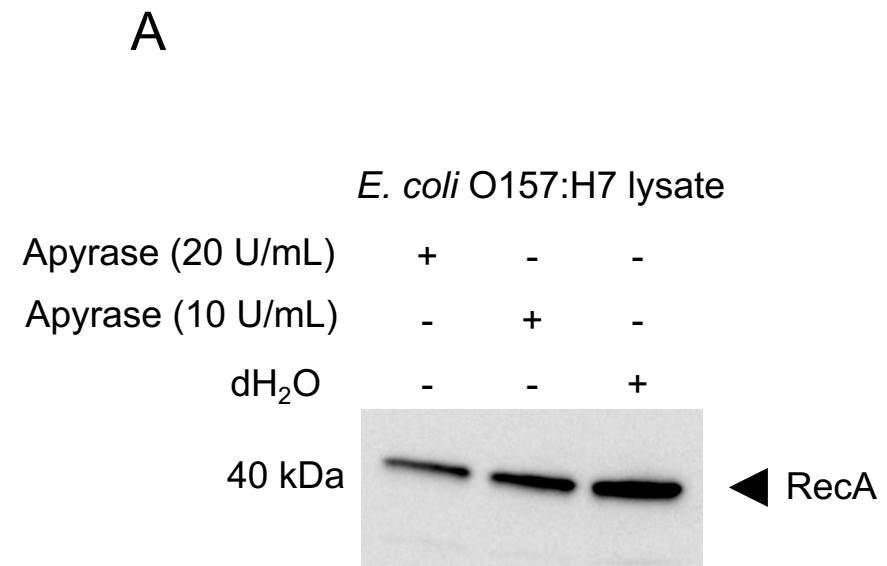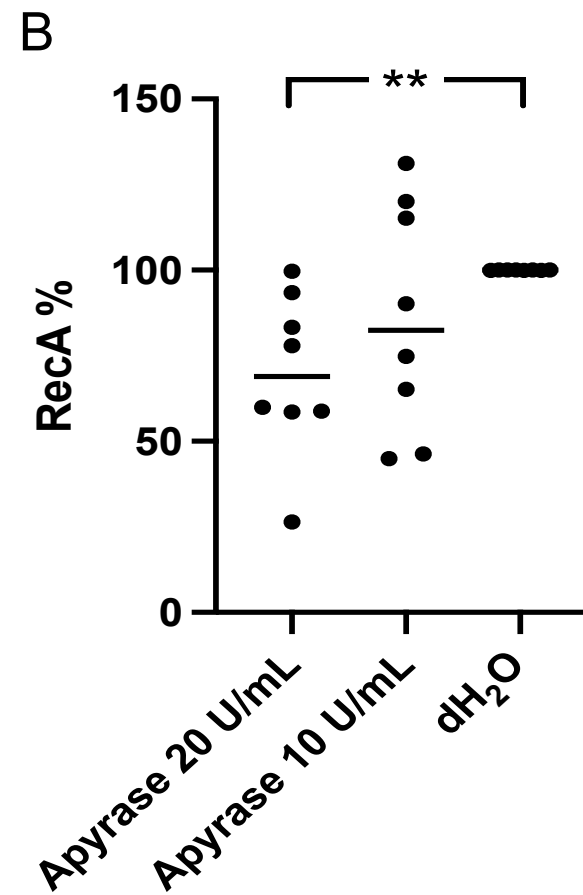

Figure 1

Supplement: Supplemental Material [file KGMI_A_2122667_SM5382.zip › Supplementary Figure 1.pdf]

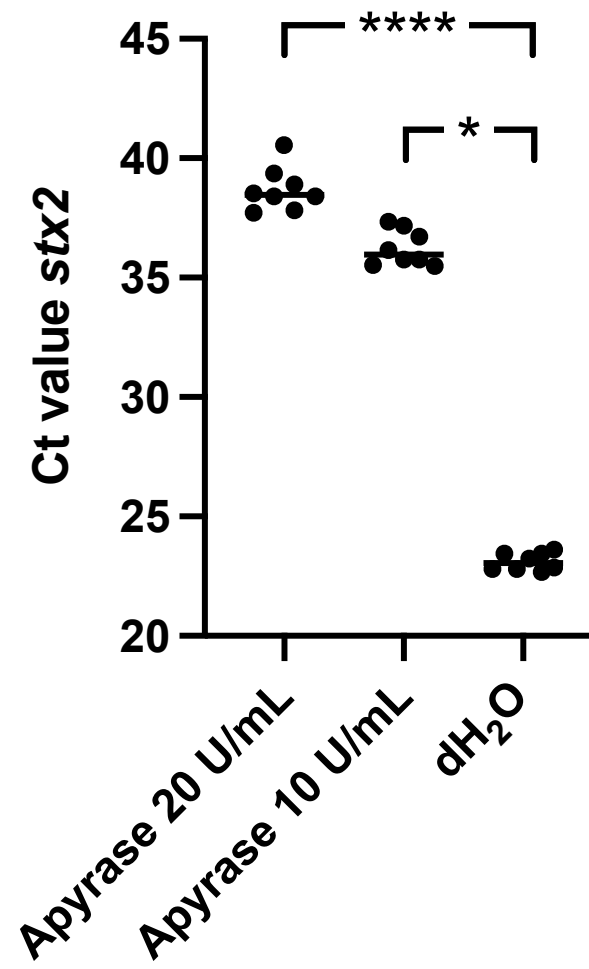

Figure 2

Supplement: Supplemental Material [file KGMI_A_2122667_SM5382.zip › Supplementary Figure 2.pdf]

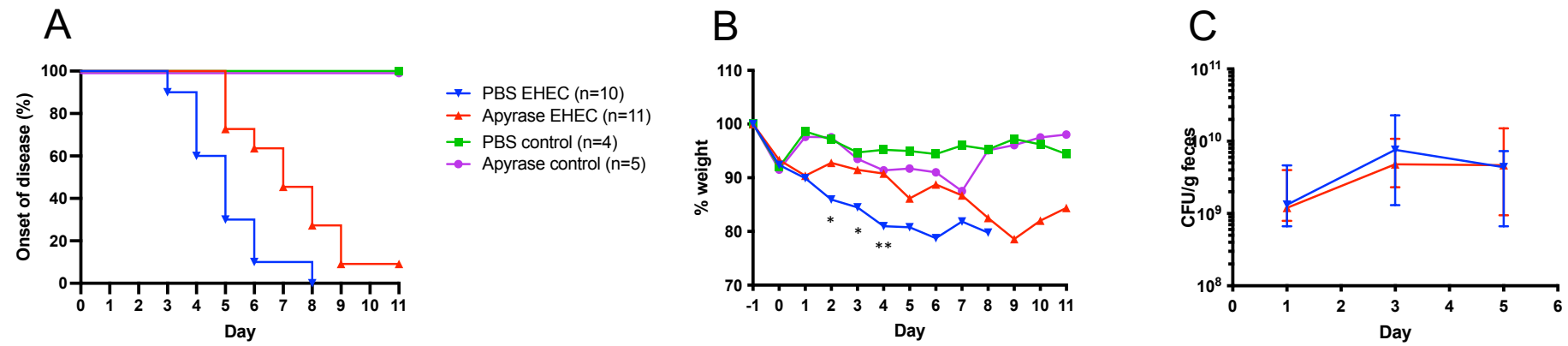

Figure 4

Supplement: Supplemental Material [file KGMI_A_2122667_SM5382.zip › Supplementary Figure 4.pdf]

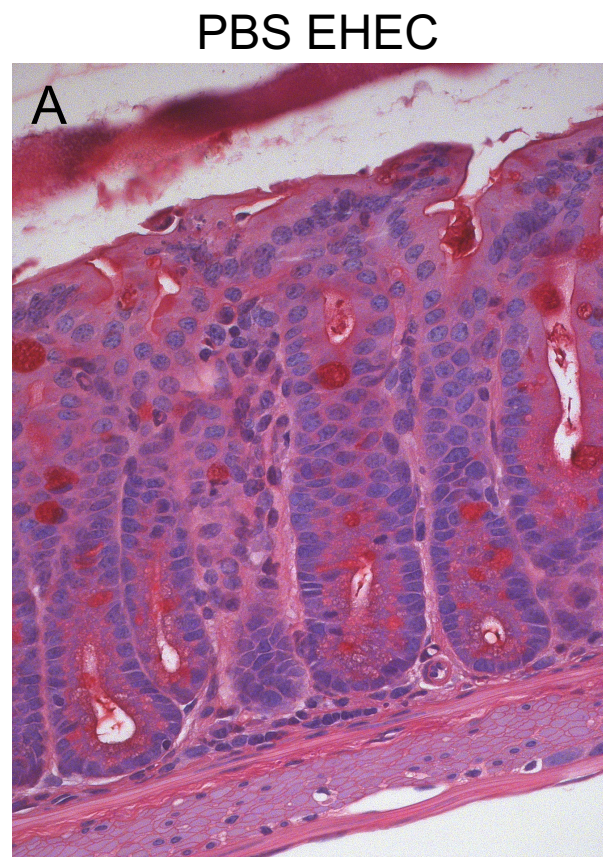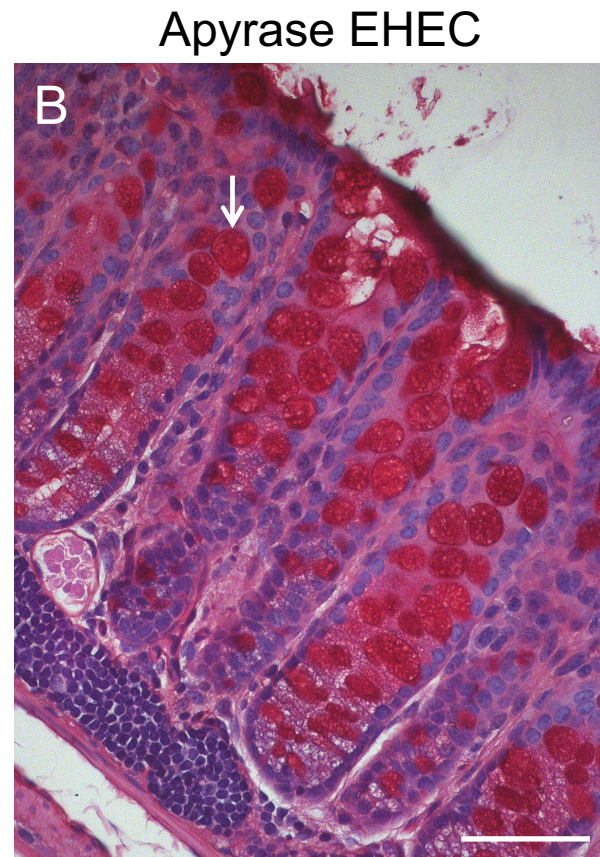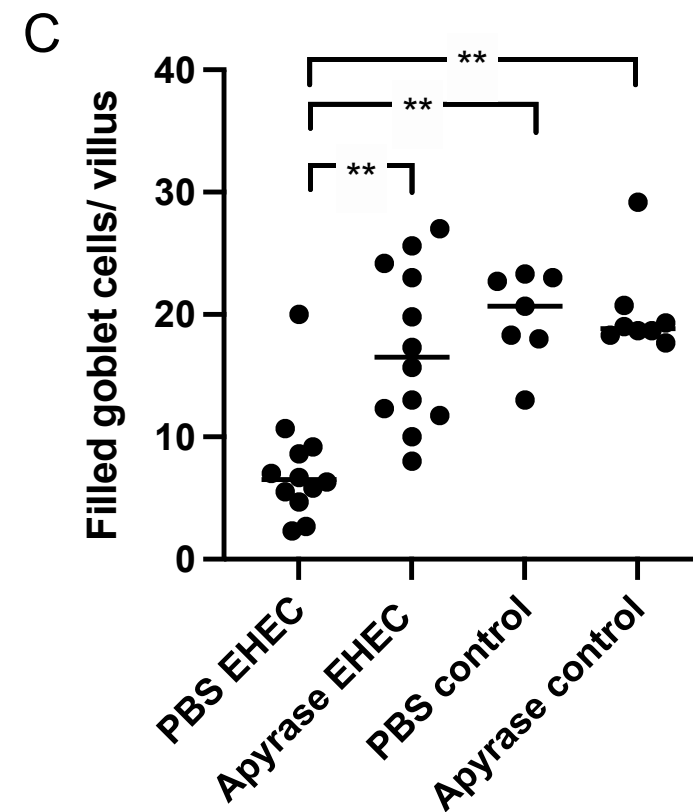

Figure 5

Supplement: Supplemental Material [file KGMI_A_2122667_SM5382.zip › Supplementary Figure 5.pdf]

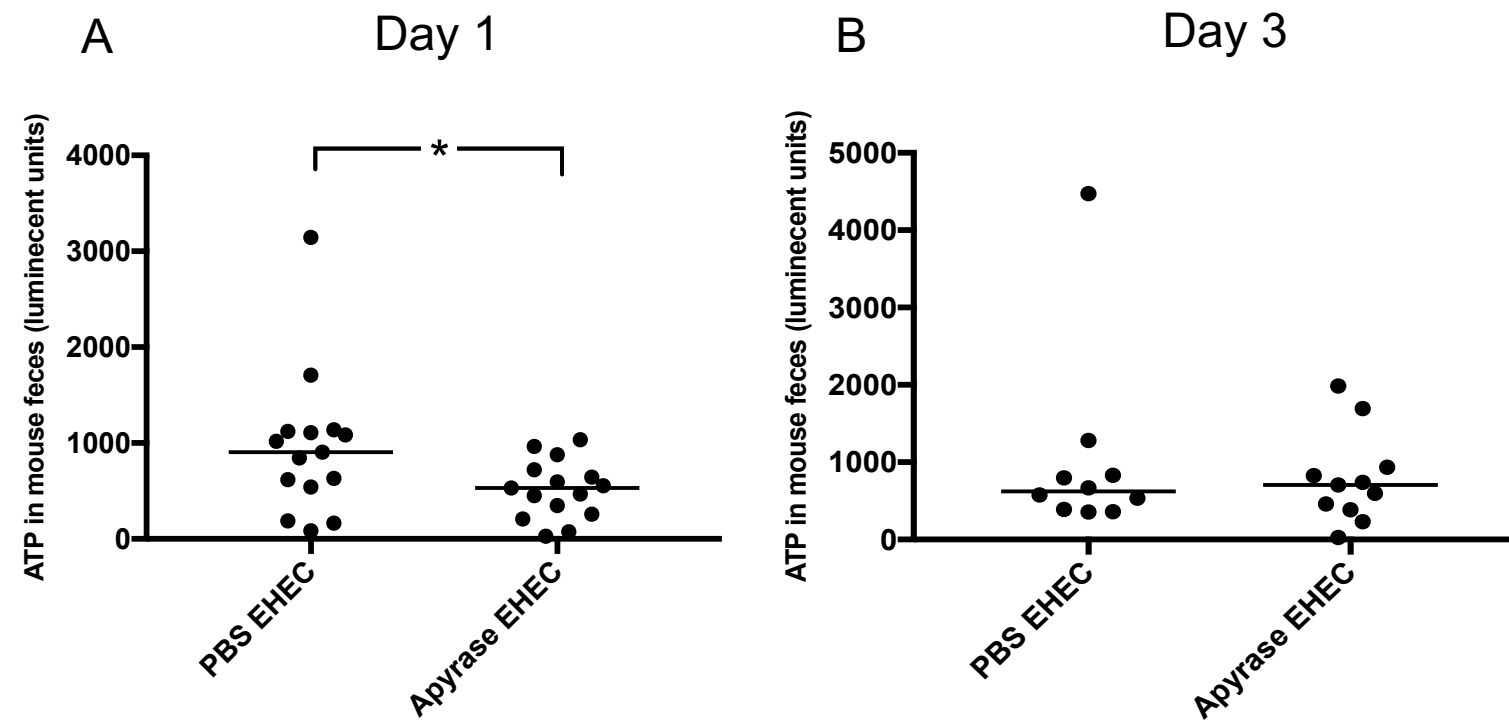

Figure 7

Supplement: Supplemental Material [file KGMI_A_2122667_SM5382.zip › Supplementary Figure 7.pdf]
